# The Dilemma of Medical Reimbursement Policy in Rural China: Spatial Variability between Reimbursement Region and Medical Catchment Area

**DOI:** 10.3390/ijerph16162867

**Published:** 2019-08-10

**Authors:** Yongqing Dong, Liping Fu, Ronghui Tan, Liman Ding

**Affiliations:** 1 College of Management and Economics, Tianjin University, Tianjin 300072, China; 2 Center for Social Science Survey and Data, Tianjin University, Tianjin 300072, China

**Keywords:** Medical reimbursement, spatial accessibility, rural residents, Hubei Province

## Abstract

Since the initiation of the New Rural Cooperative Medical Scheme (NCMS) in 2003 in China, medical reimbursement plays an increasingly important role in reducing the familial burden of critical illness healthcare in rural China. However, the current medical reimbursement system is operated based on prefecture-level administrative boundaries, which may prevent some residents from accessing higher-quality medical resources. Using a reliable and high-accuracy geographic information system (GIS) dataset, this study investigates whether this reimbursement system restricts rural residents from freely seeking out medical services in the Hubei Province by employing a two-step floating catchment area (2SFCA). Results show that there are spatial differences between the catchment area of different graded medical centers and prefecture-level administrative boundaries. Spatial reimbursement boundaries should be readjusted so that most rural residents receive equitable coverage by the system and reimburse their medical expenses in a more convenient way. Therefore, we argue that the local government should delineate the spatial region of the medical reimbursement for rural residents according to an assessment of their spatial accessibility to different graded medical centers beyond prefecture-level boundaries. We also discuss potential methods for designing reimbursement boundaries and reimbursement management strategies that the Chinese central government could adopt.

## 1. Introduction

Since the initiation of the New Rural Cooperative Medical Scheme (NCMS) in 2003, China made great progress in healthcare during this medical reform period and generated extensive literature on evaluating the impact of NCMS on the wellbeing of rural individuals. Several studies argued that the impact of the NCMS is still limited [1,2,3]. Their results showed that the NCMS neither decreases out-of-pocket expenditure nor increases utilization of formal medical services or improve health status, as measured by self-reported health status and sickness or injury in the past four weeks. However, many more studies showed that NCMS achieved great success [4,5,6,7,8,9]. For example, one study showed that NCMS provides financial risk protection for individuals in rural China and partly corrected distortions in Chinese rural healthcare by reducing the oversupply of specialty services and prescription drugs [6].

**Figure 1:**
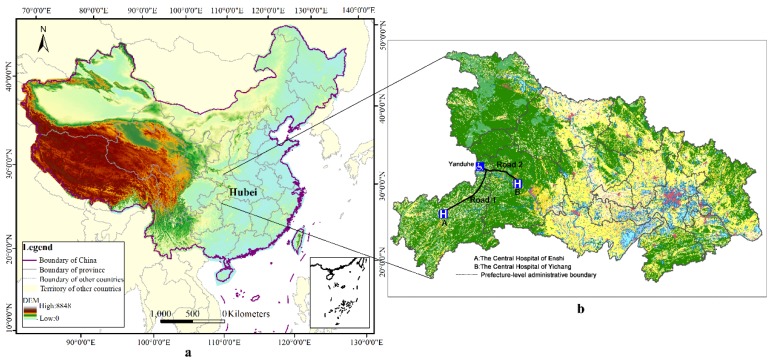
The location of Hubei Province in China (**a**), and one example of transborder medical seeking behavior that leads to reimbursement inconvenience (**b**).

Although healthcare reform in rural China was fruitful, some problems still persist. One of the most striking problems is reimbursement inconvenience caused by spatial differences between the catchment area of different graded medical centers and prefecture-level administrative boundaries. According to the requirements of the “unified fundraising standards, unified benefit policies, unified management procedures, and unified information systems”, rural residents’ medical insurance should be managed under the authority of prefecture-level administrative areas in accordance with the principles of “budget management, fund adjustment, and risk sharing” [10]. Under the current management system, individuals can only reimburse their medical expenditure if they seek medical care from hospitals located within the prefecture-level administrative area which their registered permanent residence belongs to [10,11]. However, to a large extent, the spatial or geographic distribution of hospitals does not match with the prefecture-level administrative area divisions in China. Hospitals are unevenly distributed across the province, prefecture-level administrative areas, and rural residential points [12,13,14]. Such unequal access may underlie the reimbursement inconveniences that rural individuals face, particularly individuals who require medical care from Grade-A or Grade-B hospitals. To shed light on the relationship between reimbursement inconvenience and administrative division, we assume individual *i* lives in a town named Yanduhe, located in the north-eastern region of Enshi, a prefecture-level administrative area in the southwest of Hubei Province (Figure 1b). The town is approximately 246 km away from the Central Hospital of Enshi which is the designated hospital for healthcare insurance and at the administrative center of Enshi—approximately six hours to the hospital by highway. Yet, the Central Hospital of Yichang, located in Yichang, a different prefecture-level administrative area in Hubei Province, is much closer (approximately 177 km) to individual *i*. The distance between the home of individual *i* and the Central Hospital of Yichang is approximately 177 km—only requiring a minimum of three hours by highway. This discrepancy primarily applies to individual *i* who resides in the region bordering Enshi and Yichang, such that, when individual *i* is seriously ill, the best choice is to seek medical care from the Central Hospital of Yichang. Yet, individual *i* can only reimburse his medical expenditure if he seeks medical care in the Central Hospital of Enshi and cannot reimburse his expenditure if he seeks medical care in the Central Hospital of Yichang. This example is somewhat popular around the province. There are a large number of rural residents that feel that the reimbursement procedure is extremely inefficient and bureaucratic in the administration of rights. This inconveniences rural residents and wastes medical resources.

There are a few studies that touched upon the problem raised here. One explored the impact of the border between Haiti and the Dominican Republic on access to health resources, and found that border openness had great impact on the spatial access to healthcare for the population living next to the border and those living nearby a road network in good condition [15]. Hu and his co-authors assessed the potential spatial accessibility of health services in rural China using Donghai County as a case study [16]. Another study measured and analyzed spatial accessibility to medical services for elderly people in Wuhan [17]. Yet, there still exist some gaps in the relevant studies mentioned above. Firstly, they did not touch upon the problem of reimbursement convenience when they investigated spatial accessibility. Secondly, the samples were limited in the two studies, which took some specific area of China as the research background. For example, the last study mentioned above focused only on elderly people and used Wuhan (a city in China) as the research area [17]. More importantly, researchers are yet to investigate the problem at the provincial level, a key aspect of understanding the reimbursement inconvenience of rural residents. Lastly, these studies did not distinguish between different grade hospitals, treating them as homogeneous.

Measuring spatial accessibility received increasing attention in recent years. This study is arguably of some importance because it measures the medical accessibility of rural areas and investigates the rationality of a medical reimbursement system divided by administrative areas by investigating reimbursement inconvenience in rural Hubei Province. Our findings have implications for reimbursement policies concerned with geographic adjustment and regional cooperation between different administrative areas caused by transregional medical seeking behavior. As far as we know, there are no any studies that touched upon medical reimbursement in rural China on the basis of spatial accessibility, with most prior studies tending to solely focus on medical accessibility as mentioned above. Therefore, the overall aim of this study was to investigate the relationship between medical spatial accessibility and reimbursement convenience in the rural Hubei Province. To achieve this aim, this study formulated three specific objectives: firstly, to calculate the medical accessibility for each residential point; secondly, to compare the distribution of medical spatial accessibility and residential point across different regions; and last but not least, to depict the regions characterized by rural resident reimbursement inconvenience and gauge its proportion. This study, to the best of our knowledge, makes at least three contributions to the literature. Firstly, we verify that the spatial accessibility to hospitals is not consistent with the administrative division in rural Hubei Province. Secondly, we use high-resolution geographic information system (GIS) data, combining web data capturing the special accessibility to two different kinds of hospitals. Last but not least, we analyze the impact of administrative division on the reimbursement inconvenience with a perspective of spatial accessibility, and depict these rural regions which are featured by reimbursement inconvenience. Despite the contributions mentioned above, we acknowledge at least two limitations of our study. Firstly, due to the data limitation, we use straight-line distance rather than the road network distance, and this may cause bias to some extent. Secondly, the conclusion of this study depends on a hypothesis which seems to be a little strong. In this study, the most important factor that has impact on the decision of hospital choice when individuals seek medical care service is spatial accessibility. We do not consider the impacts of other factors, such as income, education background, etc., on the choices of rural residents. In a further study, we will try to cope with these problems by searching for more reliable data and using advanced approaches.

The rest of this study is structured as follows: in the next section, we describe the study area and data used in this study firstly, then make a brief introduction of the method. Section 3 presents our empirical results. Section 4 comprises a discussion on the method and results of this study. The summary of the study’s findings is presented in the final section. 

## 2. Materials and Methods 

### 2.1. Study Area

Our study focuses on the Hubei Province located in the central China. It is situated between longitude 108’21” east (E) and 116’07” E, and latitude 29’05” north (N) and 33’20” N. Hubei is one of the six provinces of central China (i.e., Shanxi, Henan, Anhui, Jiangxi, and Hunan) and covers an area of 185,897 square kilometers. The provincial capital is Wuhan, a major transportation, political, economic hub of central China. Hubei Province consists of 13 prefecture-level areas (including 12 prefecture-level cities and one autonomous prefecture-level area) that contain 103 county-level administrative areas. Furthermore, Hubei Province also includes three sub-prefecture cities and one forest region. As such, although we set the number of prefecture-level administrative areas in Hubei Province to 13 when we computed the service radius of hospitals, we still produced detailed descriptive statistics for the three sub-prefecture cities and one forest region.

The primary reason for selecting Hubei as the study area was because the medical accessibility of rural residents in Hubei is unequal, particularly for citizens who reside by the prefectural border area. In 2015, Hubei Province reported a total population of 58.52 million (with 29.85 million males (51.01%) and 28.67 million females (48.99%)). Among the residential population, approximately 33.27 million (56.85%) reside in urban areas, while about 25.25 million (43.15%) live in the rural areas, reflecting a relatively smaller share of the population. However, residents living in rural areas are more likely to face limited access to medical resources and inconvenient reimbursement. Geographically, the terrain of Hubei Province is relatively high in the west and low in the east. The Jianghan Plain takes up most of the central and southern area, while the west and peripheries consist of more mountains, such as Shennongjia in the west, Dabie Mountain in the east, and Wudang Mountain in the northwest. The altitudes of these mountains are over 1000 meters. The highest peak (Shennong Peak) has an altitude of 3105 m. These mountains also represent a barrier to rural residents’ access to medical resources in and out of Hubei Province. Therefore, in this study, we demonstrate why some rural residents seek medical resources across prefectural borders and waive reimbursement opportunities.

Thus, we selected Hubei as study area because of the advantage of its location in this paper. In the majority of cases, citizens who live near the outer border of the province probably seek care outside the province more than anyone else in China. Yet, most rural people may not choose to seek care outside of Hubei in this study. On the one hand, individuals cannot reimburse their expense if they seek care outside the province. On the other hand, such individuals find seeking care relatively inconvenient if they seek care outside the province due to Hubei being surrounded on three sides (east, west, and north) by several mountains. These mountains relatively lower the medical accessibility of residents in rural Hubei Province to those hospitals located in neighborhood provinces, such as Shaanxi Province and Anhui Province. Hence, the results of analysis in this study would be immune from the spillover effect of hospitals in the neighborhood of Hubei Province to a large extent. However, we still acknowledge that we underestimated reimbursement inconvenience to some extent.

### 2.2. Data Sources and Pre-Processing

**Figure 2:**
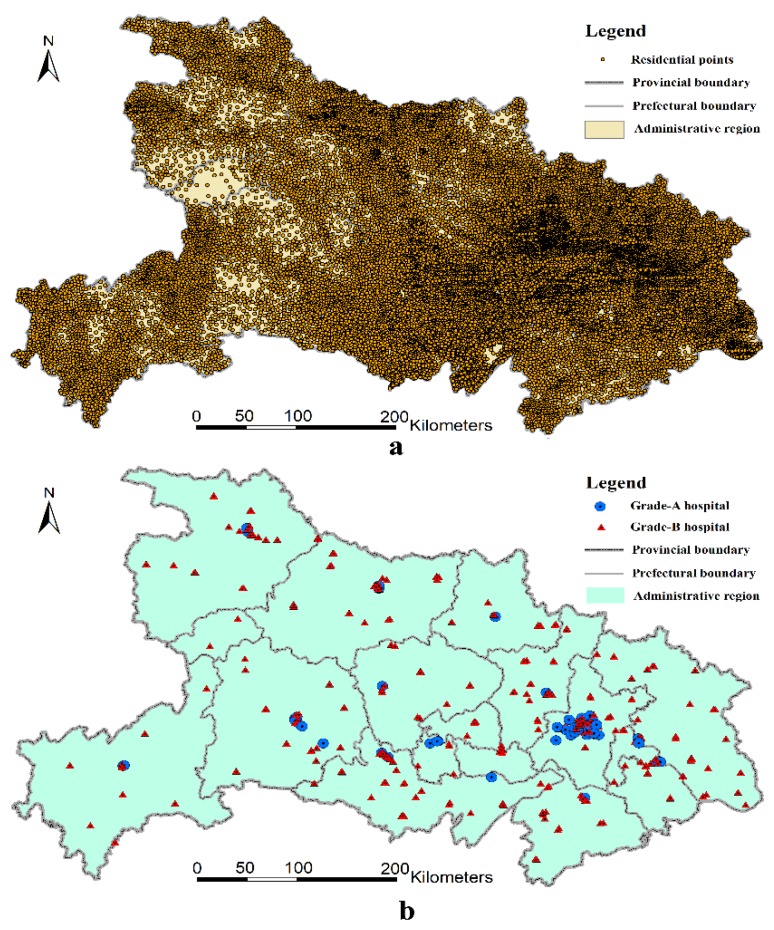
The geographical distribution of residential points (**a**), and the geographical distribution of Grade-A and Grade-B hospitals in Hubei Province (**b**).

The residential data used in this study are based on the land-use/land-cover (LULC) vector format maps at a 1:10,000 scale, which were gathered in the second national land-use survey (ended in 2009) conducted by the Ministry of Land and Resources of the People’s Republic of China. To obtain new information on land use for policy-making, after the first national land-use survey conducted in 1996, China initiated its second national land-use survey on 1 July 2007. Local authorities finished the survey before the end of June 2009 and submitted the new land-use data to the Ministry of Land and Resources on 31 October 2009. In this round of the survey, China used advanced remote imaging technologies to survey all land-use types, which included 12 categories (e.g., farmland, forests, residential points, business, water, etc.) and 56 sub-categories. The land-use type, area, location, and ownership of each plot were also recorded. As such, these spatial data have high spatial resolution. Based on the second national land-use survey data, we then extracted the polygon features of rural residential land and converted them to point features that represent their geographical distribution. As shown in Figure 2a, most of the rural population is concentrated in the low and flat middle part of Hubei Province. 

Finding a hospital database with spatial information is usually a challenging task in China, as hospital data are usually not released by the authorities. In order to overcome this difficulty, we used a web scrapper to derive the *X*- and *Y*-coordinates of all hospitals in Hubei Province from Baidu map based on key words, such as “sanjia”, “yiyuan”, etc. [18]. Next, we recorded the hospital name information, as hospitals differ from each other in terms of the quality, coverage, and price of service provision. Thus, it is natural to distinguish hospitals in terms of rural residents’ spatial accessibility to them. 

In China, hospitals are classified into three major classes: Grade-A hospitals (in Chinese: “Sanji” hospitals); Grade-B hospitals (in Chinese: “Erji” hospitals), and Grade-C hospitals (in Chinese: “Yiji” hospitals) [19,20]. Grade-A hospitals are first-class hospitals that own over 501 hospital beds and are equipped with the most advanced medical equipment. Grade-B hospitals provide general medical services and have 101–500 hospital beds. Grade-C hospitals are the lowest-level hospitals that provide medical services in communities and typically have fewer than 100 hospital beds [21]. We only selected Grade-A or Grade-B hospitals in this study since Grade-C hospitals could not be identified accurately on the map and are usually not the first choice of rural residents when they require and seek medical care. Grade-A hospitals can provide medical and health services across different regions and cities, and they provide coverage all across China, although most predominantly provide medical care within the prefecture-level administrative area. These hospitals are medical centers with comprehensive medical, diagnostic, teaching, and research capabilities. Their main functions involve the provision of medical services and treatment of severe disease. Most Grade-A hospitals are located in the province-level municipalities, provincial capitals, or sub-provincial cities. Meanwhile, most ordinary prefecture-level administrative areas only have access to one or very few Grade-A hospitals; some may have no access to Grade-A hospitals. Grade-B hospitals are regional hospitals that provide comprehensive medical and health services to a number of communities or townships. Most counties only contain a few Grade-B hospitals. A brief summary of different grades of hospitals can be found in Table 1.

Generally, Grade-A hospitals are the best hospitals as they are larger and have the best medical facilities, most advanced technologies, and the best management capability. They provide high-level and specialized medical and health services at a prefecture level, province level, or even national scale. Grade-B hospitals have relatively lower standards and provide services at the county level. Grade-C hospitals are basic medical institutions which provide services within a community. They usually provide healthcare services rather than medical treatment services. Note that we did not include the Grade-C hospitals in our analysis. On the one hand, the Grade-C hospitals are usually not the first choice when rural individuals seek medical care services due to their worse medical conditions. If a rural member goes to Grade-C hospitals for medical service, it usually means that his or her health is not in bad condition; on the other hand, the Grade-C hospitals were difficult to identify when using the web scrapper to extract their geographical information, as they have mixed names. To sum up, we collected the geographical information of 92 Grade-A hospitals and 313 Grade-B hospitals in Hubei Province (please see Figure 2b).

As can be seen in Figure 2b, Grade-A hospitals are mainly concentrated in Wuhan, Yichang, and Xiangyang, while Grade-B hospitals show a relatively even distribution among different prefecture-level regions. The distribution of hospitals is largely unrelated to the geographical characteristics and the distribution of residential points. This arguably leads to reimbursement inconveniences for specific portions of the population, particularly for those who live near the bordering regions between two prefecture-level administrative areas.

### 2.3. The Two-Step Floating Catchment Area Method

We calculated medical accessibility by using the two-step floating catchment area (2SFCA) method, which was first proposed by Radke and Mu to overcome the shortcomings of the traditional gravity models that measure accessibility [22]. Earlier versions of the floating catchment area were used in several fields, particularly in job–housing analysis [23,24]. This method could also be used to analyze the medical accessibility of different residential points based on a supply and demand analysis of medical care. Given that the procedure of this method needs to be implemented in two steps, it is called the two-step floating catchment area method [25].

**Step 1:** For each supply point *j*, we searched all demand points (*k*) within the distance threshold of $d_{0}$ (the search area of *j*), so we could calculate the supply–demand ratio $R_{j}$ within the catchment area.

(1) $$R_{j}=\frac{S_{j}}{\sum_{k\in \left\{d_{kj}\leq d_{0}\right\}}D_{k}},$$

where $d_{kj}$ is the distance between residential point *k* and residential point *j*, $D_{k}$ is the medical demand of each residential point in the search area ($d_{kj}\leq d_{0}$), and $S_{j}$ is the medical supply of point *j*. 

**Step 2.** For each demand point *i*, we search all supply points (*j*) that are within the distance threshold of $d_{0}$ (the search area of *i*); then, we can acquire the accessibility of point *i* by summing the supply–demand ratio $R_{j}$.

(2) $$A_{i}^{F}=\sum_{j\in \left\{d_{ij}\leq d_{0}\right\}}R_{j}=\sum_{j\in \left\{d_{ij}\leq d_{0}\right\}}\left[\frac{S_{j}}{\sum_{j\in \left\{d_{ij}\leq d_{0}\right\}}D_{k}}\right],$$

where $d_{ij}$ is the distance between point *i* and *j*, and $R_{j}$ is the supply–demand ratio of point *i*’s search area ($d_{kj}\leq d_{0}$). This indicates improved medical accessibility as $A_{i}^{F}$ increases. As mentioned previously, the superscript *F* indicates that the equation is based on the floating catchment area method.

**Figure 3:**
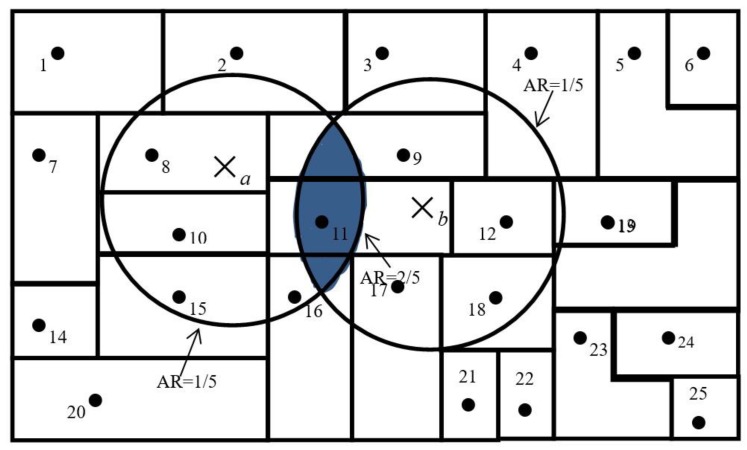
A simplified version of the two-step floating catchment area (2SFCA) method. AR = accessibility ratio.

A simplified version of the 2SFCA method is presented in Figure 3. For simplicity, Figure 3 assumes that each residential point has only one person residing at its centroid and each hospital location only has one physician. We also make the assumption that a threshold travel distance for healthcare from a hospital is 24 kilometers. A 24-km circle around the centroid of residential point 2 defines its catchment area. Medical accessibility at a specific residential point is defined as the accessibility ratio within its catchment area. The catchment area of hospital *a* has one hospital and five residential points and, thus, carries an accessibility ratio of 1/5. Similarly, the accessibility ratio for hospital *b* is also 1/5. Residential points at 8, 10, 11, 15, and 16 have access to hospital *a* only and the ratio for them remains at 1/5; residential points 9, 11, 12, 17, and 18 have access to hospital *b* only and, thus, have a ratio of 1/5. However, the residential point at 11 is located in an area that overlaps the catchment areas of hospitals *a* and *b*, and has access to both hospitals *a* and *b*; therefore, it enjoys better accessibility (that is, a higher ratio of 1/5 + 1/5 = 2/5). The overlapping area was identified in the second step, through which it was identified that hospitals *a* and *b* were both within a 24-kilometer catchment area of residential point 11.

As a special case of the gravity model, the 2SFCA method is more intuitive to interpret and was used in a number of recent studies, particularly those that focused on healthcare accessibility [26,27,28,29]. We employed the 2SFCA method in this study since it is still one the most popular and scientific methods to measure the accessibility of medical services. As mentioned above, it could be used to compute the ratio of hospitals to residential points within a service area centered at a hospital’s location, and it sums up the ratios of residential points located in areas where different hospitals’ services overlap with each other. This serves to address the problem whereby medical services that fall within the catchment area are fully available to residential points within that catchment area [25].

### 2.4. Defining the Service Radius

Considering that Grade-A and Grade-B hospitals differ from each other in terms of size and quality of services, their different service radii should be defined from the onset. Previous studies used a travel time threshold (10 min and 60 min) to represent service radius in 2SFCA [17,30]. A travel time threshold refers to the waiting time before one can receive advanced medical treatment in the case of an emergency. Yet, our primary focus was not on how quickly but how conveniently residents could acquire healthcare. Although less travel time is an indicator of increased convenience, many rural residents would not mind the time expended traveling to acquire medical services. In reality, shorter distances are more important for rural residents traveling in the west and east mountainous areas of Hubei Province. Therefore, we used a distance threshold to represent service radius in this study. As the main function of Grade-A hospitals is to provide medical services within the prefecture-level administrative area, while the main function of the Grade-B hospitals is to provide medical services within the county-level administrative area, we set the service radius for each based on the average radius of prefecture-level and county-level administrative areas. Hubei Province consists of an area of 185,897 square kilometers and 13 prefecture-level administrative areas. The service radii of Grade-A hospitals were set by equating them with the average radius of each prefecture-level administrative area in the Hubei Province, that is, 67.47 kilometers (=$\sqrt{185,897/(13\times \pi )}$). Similarly, we set the service radii of Grade-B hospitals to equal the average radius of county-level administrative areas, that is, 23.97 kilometers (=$\sqrt{185,897/(103\times \pi )}$). Based on these two radii, we employed the 2SFCA method to calculate the medical accessibility ratios for every rural residential point in Hubei Province.

## 3. Results

### 3.1. Statistical Analysis of Reimbursement Accessibility

**Figure 4:**
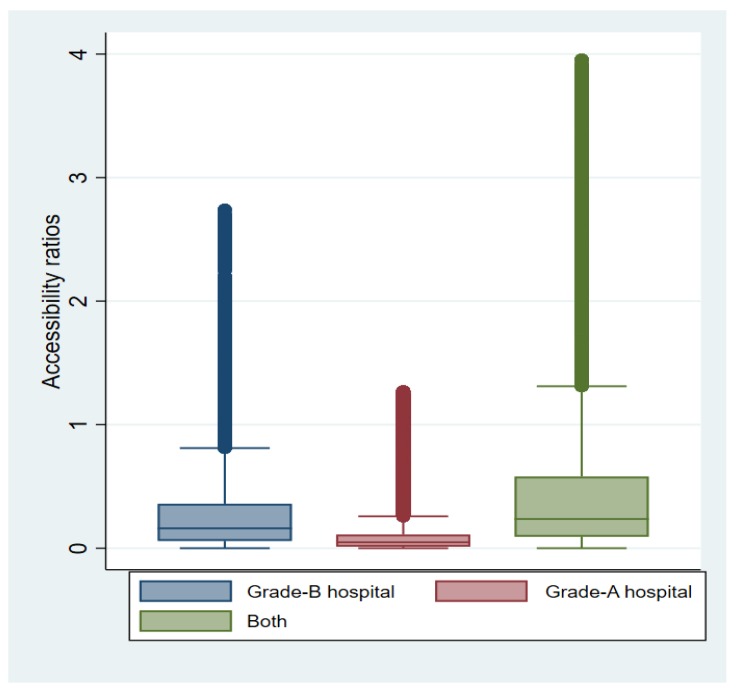
Variation in accessibility ratios of rural residents in Hubei Province.

We computed the medical accessibility ratios of 1,320,438 residential points from 30,187 villages in Hubei Province. The medical accessibility ratios among different residential points showed significant heterogeneity (Table 2). For Grade-A hospitals, the average value of medical accessibility ratios was 0.22, while the lowest medical accessibility ratio among the residential points was 0 and the highest was 1.27. For Grade-B hospitals, a similar story was revealed. When we simultaneously considered both Grade-B and Grade-A hospitals, the mean of medical accessibility ratios was 0.52 and significant medical accessibility heterogeneity among different residential points was observed. The box plots of the accessibility ratios of Grade-A, Grade-B, and both hospitals are depicted in Figure 4. Overall, the findings indicate that medical accessibility to hospitals is highly unequal for rural residents in Hubei Province. 

We further investigated the percentile distribution of the medical accessibility ratios of Grade-A, Grade-B, and both hospitals. Again, the heterogeneity of medical accessibility ratios was clear, with over 10% of residential points receiving a score of 0 for Grade-B hospitals, while almost 25% reported a 0 score for Grade-A hospitals. A 0 medical accessibility score indicates extremely scarce or completely inaccessible health and medical services. At the Grade-A hospital level, over 75% of residential points had medical accessibility ratios below average. When we investigated the percentile distribution of medical accessibility by taking both the Grade-B and Grade-A hospitals into account, the average medical accessibility ratio was almost at the 75th distribution percentile. In sum, these results also show significant inequality in access to medical services among different rural residential points in Hubei Province.

Considering the regional heterogeneity, we computed the medical accessibility ratios for every prefecture-level administrative area according to Grade-A and Grade-B hospitals (Table 3). At the Grade-A level, we found that Enshi and Shenlongjia had relatively lower medical accessibility ratios, i.e., 0.01 and 0, respectively. Contrastingly, Ezhou and Wuhan had relatively higher medical accessibility ratios, which were 0.93 and 1.12, respectively. At the Grade-B hospitals level, we could see that Enshi and Yichang had relatively lower medical accessibility ratios, i.e., 0.02 and 0.06, respectively. Ezhou and Wuhan had relatively higher medical accessibility ratios, which were 0.76 and 0.78, respectively. When combining all Grade-B and Grade-A hospitals together, the results showed that Enshi and Yichang had relatively lower medical accessibility ratios, i.e., 0.02 and 0.10, respectively, while Ezhou and Wuhan still had relatively higher medical accessibility ratios, i.e., 1.70 and 1.90, respectively. Numerous studies showed that there is significant regional inequality in medical resources in China. These findings are in line with those results and demonstrate healthcare (accessibility) inequality in rural Hubei Province.

### 3.2. Spatial Variation of Reimbursement Inconvenience Regions

**Figure 5:**
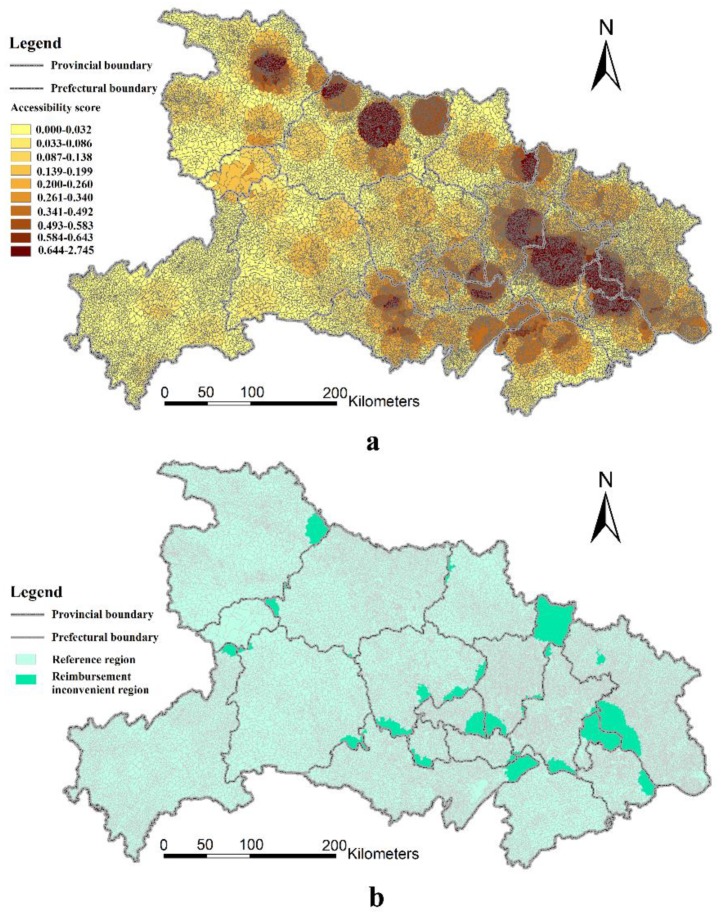
Accessibility ratios to Grade-B hospitals (**a**), and the geographical distribution of reimbursement inconvenience regions with respect to Grade-B hospitals (**b**).

Figure 5a shows the spatial variation of medical accessibility ratios in Hubei Province at the Grade-B hospital level using the 2SFCA method. One prefecture-level administrative area in particular enjoys the best access, namely, Wuhan—the capital city of the Hubei Province and a major city in central China. Wuhan owns a large number of hospitals including a large number of Grade-B hospitals. Meanwhile, aside from Wuhan, the results show several prefecture-level administrative areas also enjoy good accessibility, such as Xiangyang and Ezhou. Xiangyang is recognized as the political, economic, medical, and educational center in the northwest region of Hubei Province. Contrastingly, Ezhou, a neighboring city of Wuhan, has a number of hospitals and a relatively small rural population. Since the administrative centers of prefecture-level administrative areas are also the economic, medical, and educational centers for the area, it is clear that the regions directly surrounding the administrative center of each prefecture-level administrative area enjoy better access to healthcare. However, most rural areas that are located far from the administrative center in Hubei Province suffer from insufficient access.

Based on Figure 5a, the distribution of reimbursement inconvenience regions in rural Hubei Province is depicted in Figure 5b, which shows that the reimbursement inconvenience regions are mostly located in areas bordering the two prefecture-level administrative areas. These regions include the northeastern regions of Xiaogan, the southwestern regions of Huanggang, the eastern regions of Shiyan, the northeastern regions of Jingzhou, and so on. Overall, most of these regions are located in prefecture-level administrative areas which are neighborhoods of Wuhan. As such, it is more convenient for residents in these regions to seek medical care in Wuhan. However, if they choose to acquire medical services in Wuhan, they cannot reimburse their expenditure in their residing prefectural regions. This situation depicts a classic example of the dilemma of medical reimbursement policy in rural China. 

**Figure 6:**
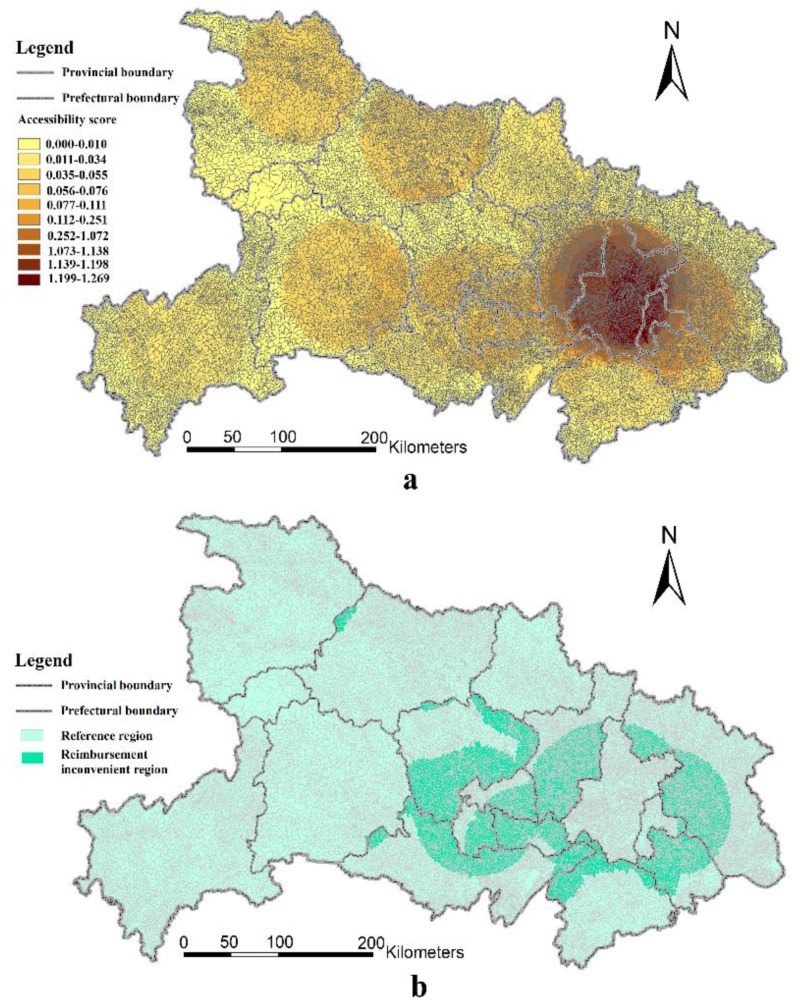
Accessibility ratios to Grade-A hospitals (**a**), and the geographical distribution of reimbursement inconvenience regions with respect to Grade-A hospitals (**b**).

Figure 6a depicts the spatial variation of medical accessibility ratios of Grade-A hospitals in the Hubei Province using the 2SFCA method. Here, Wuhan still enjoys the best accessibility. This result is consistent with the results of Grade-B hospitals mentioned above. Moreover, we did not find that the prefecture-level administrative areas outside of Wuhan enjoyed good accessibility from Grade-B hospitals either. Thus, there seems to be a large difference in the medical accessibility ratios of rural residents between Wuhan and non-Wuhan areas. This is because the majority (over 50%) of Grade-A hospitals are located in Wuhan. Although prefecture-level administrative cities are regarded as their own medical centers for each prefecture, only a few have Grade-A hospitals. In fact, rural areas in Hubei Province suffer from insufficient medical accessibility to Grade-A hospitals, indicating that high-quality medical resources are concentrated highly in Wuhan.

Similar to Figure 5b, Figure 6b depicts the distribution of reimbursement inconvenience regions in rural Hubei Province based on the results of medical accessibility to Grade-A hospitals. Consistent with the results of Grade-B hospital medical accessibility analysis, it also shows that the reimbursement inconvenience regions are mostly located in these border regions between two prefecture-level administrative areas. However, compared with the medical accessibility to Grade-B hospitals, there are two interesting findings in the analysis of medical accessibility to Grade-A hospitals. On the one hand, the total area of reimbursement inconvenience regions is much larger than that of Grade-B hospitals in Figure 6b. On the other hand, the inconvenience regions, with respect to medical accessibility to Grade-A hospitals, are much more concentrated, and most of them are located in the surrounding area of Wuhan. In other words, a great number of residents from the neighborhood prefectural-level regions of Wuhan who reside around the boundary of Wuhan city may obtain better medical services. However, the current reimbursement policy restricts them from acquiring advanced medical services from Wuhan because they cannot refund their medical expenditures.

Table 4 presents the descriptive statistics of the percentage of reimbursement inconvenience regions based on prefecture-level administrative areas. At the Grade-B hospital level, 7.79% of rural residents suffer from reimbursement inconvenience, which is the equivalent of 3.34% of residential points in rural Hubei Province. Most prefecture-level administrative areas face the problem that their rural residents can only refund their medical expenditures in their residential prefectural-level administrative areas. This division leads to circumstances that inconvenience rural residents seeking medical care. For example, 17.66% of villages in Xiaogan suffer from reimbursement inconvenience, and this accounts for 22.4% of the rural residents in this city. The results also show that the prefectural regions with reimbursement inconvenience issues are located predominantly in regions bordering administrative areas. As such, many have large administrative areas but relatively small urban areas and a limited number of Grade-A hospitals. Therefore, rural residents in these prefectural regions are more likely to cross these borders to seek medical services in neighboring prefectural regions that are more accessible.

At the Grade-A hospital level, reimbursement inconvenience is even much more apparent. Overall, 28.62% of villages suffer from the reimbursement inconvenience, which is the equivalent of 13.78% of residential points in the rural Hubei Province. For the rural residents living in these prefecture-level administrative areas, citizens have to waste a lot of money and time to access the medical care in Grade-A hospitals. Indeed, relative to the results of Grade-B hospitals, there are some significant differences. Firstly, many more prefecture-level administrative areas face reimbursement inconvenience issues. Secondly, these prefecture-level administrative areas characterized by reimbursement inconvenience are at a high risk of a more serious situation in the future. For example, over 50% of rural residents suffer from reimbursement inconvenience in Xiantao, Huangshi, Jingmen, Tianmen, and Qianjiang. Lastly, these five administrative areas—Xiantao and Huangshi in particular—showed large differences in reimbursement inconvenience between Grade-A and Grade-B hospitals.

## 4. Discussion

Overall, this study demonstrates that accessibility to healthcare from hospitals for rural residents in the Hubei Province is highly unequal, consistent with prior studies [31,32,33,34]. Furthermore, this study also verifies the existence of significant regional inequality in the distribution of medical resources in China consistent with research in related literature [35,36,37,38]. One factor that likely determined the results is the fact that the distribution of hospitals is largely not related to the actual geographical characteristics and residential point distribution. Indeed, numerous studies showed that the administrative center of prefecture-level administrative areas is also economic, medical, and educational center in the respective area [39,40]. However, most rural residents do not live in said centers.

Equitable and fair opportunity to access medical services is one of the key objectives of China’s medical reform [41,42,43,44]. Moreover, reimbursement convenience is arguably an important safeguard condition to achieving equal access to basic healthcare services. Geographical imbalance and a shortage of medical institutions in rural areas is one of the main obstacles to ensuring that healthcare is equitably distributed to all citizens of China. This is part and parcel of what underlies the inconvenience that rural residents experience in relation to reimbursing their medical expenses. As the largest developing country, rural areas in China still face the problem of reimbursement inconvenience when seeking care from their preferred hospitals. This is particularly pertinent for citizens who reside by prefectural boundaries, as their preferred hospitals may be located in another prefecture. In fact, previous studies demonstrated that a considerable portion of the rural population has poor access to healthcare services in other developing countries [44,45,46,47,48,49]. The governments of these developing countries and international organizations (i.e., World Bank and the United Nations) also invested money to address healthcare problems and shortages. Such nations may face a similar problem if these states or international organizations wish to lower the medical expenditure of residents by using medical insurance or similar mechanisms. As such, this study serves as a lesson in improving the functionality of reimbursement systems in other developing countries. Subsequently, we discuss what potential measures can be taken to make medical reimbursement more convenient in China.

### 4.1. Selecting a Method to Identify the Spatial Accessibility of Medical Services

The 2SFCA method is an important and popular method for measuring spatial accessibility to public services, which was widely applied in studies on the spatial layout of public service facilities. Many researchers extended the 2SFCA to achieve methodological advancements, such as Generalized 2SFCA, Enhanced 2SFCA, Kernel 2SFCA, and so on. Wang (2012) proposed a generalized 2SFCA by including a distance decay function in the 2SFCA model to reflect the impact of distance difference on the accessibility ratio [50]. Luo (2009) presented an enhancement of the 2SFCA (E2SFCA) method for measuring spatial accessibility, addressing the problem of uniform access within the catchment by applying weights to different travel time zones to account for distance decay [30]. Dai et al. (2011) advanced the popular 2SFCA method by incorporating a kernel density (KD) function to form the “KD2SFCA method” [51]. The advantage of E2SFCA and KD2SFCA is that distance decay effects are taken into consideration in both steps, which has solid theoretical foundation in a gravity model. Indeed, there are numerous research methods that aimed to deal with the problem of distance decay coefficients. To reduce the impact of distance decay effects on spatial accessibility, there are also many studies which took distance decay into account. However, all these studies aimed at modeling different distance impedances within the catchment area. To the best of our knowledge, there is no consistent coefficient that can be used for different regions. Therefore, the primary difficulty of using E2SFCA and KD2SFCA is having to determine an appropriate impedance coefficient. However, if local governments use these methods to determine the spatial reimbursement region, selecting an appropriate impedance coefficient will increase the difficulty of policy implementation. Another problem is that the distance decay function may not represent the real distance decay of spatial accessibility. Compared with E2SFCA and KD2SFCA, the original 2SFCA has the advantage of simple calculation. When a mass of residents makes modeling a network analysis impossible, the original 2SFCA can also be used to identify the spatial reimbursement region by local governments. If local governments have highly accurate topology data, E2SFCA and KD2SFCA using road network distances may represent a better choice.

### 4.2. Administrative Hierarchy: How to Define the Basic Spatial Reimbursement Unit

In response to the fact that the current basic spatial reimbursement unit is based on the prefectural-level administrative region, which results in reimbursement inconvenience for rural residents in Hubei, policy needs to be implemented or adjusted to deal with this dilemma. Overall, the most efficient way to deal with this problem is for the Chinese central government to give the rural residents the right to select their designated hospitals freely and reimburse their medical expenses in the hospitals they choose instead of restricting their reimbursement region to the hukou system. Using this policy, the rural residents could select hospitals they personally perceive as the most convenient, but this method may not be accepted as it is unregulatable. If this strategy cannot be accepted by the central governments, it may be better to delimit acceptable and scientific reimbursement regions based on spatial accessibility and the supply and demand of hospitals. Our results suggest that the local government could employ the 2SFCA method used in this study to plan the basic reimbursement unit based on the accessibility of rural residents. One problem that policy-makers ought to deal with in delimiting the reimbursement region is by defining the basic spatial reimbursement unit that reimbursement regions are aggregated on. In China, there are five types of hierarchical administrative units, namely, provinces (autonomous regions and municipalities directly under the Central Government, Sheng/Zizhiqu/Zhixiashi in Chinese), cities (autonomous prefectures, counties, autonomous counties, zizhizhou/Xian/Zizhixian in Chinese), counties (Xian/Qu, in Chinese), townships (Zhen/Xiang/Jiedao, in Chinese), and villages [52]. Province is the highest level of administrative unit, followed by city and county. Township is the lowest administrative unit level, and village is the lowest management unit. These two levels do not have policy-making and legislative powers. Therefore, there are only four choices in terms of defining the basic reimbursement unit. In other words, the reimbursement region can be aggregated based on villages, towns, counties, or cities. In our study, we delimited the basic reimbursement region based on administrative villages. One advantage of using villages as the basic unit is that the boundary of reimbursement region is highly consistent with the boundary of the spatial accessibility classification region. However, one disadvantage is that the reimbursement regions have a greater chance of being present near an administrative boundary, leading to inconsistent jurisdictions. Given the balance between jurisdiction and accessibility consistency, the best way to select the basic spatial reimbursement unit is by delimiting reimbursement regions using all types of administrative units, and then selecting the basic unit that generates the most consistency between reimbursement region boundaries and administrative boundaries. 

In addition, the findings of this study point to the need for efficient trans-border cooperation between health management institutions and related hospitals. One possible pathway for the current reimbursement system is to integrate several of the prefecture-level administrative areas which are reciprocal in terms of medical reimbursement together as one reimbursement region rather than two discrete reimbursement regions. In fact, a more forward-looking and radical measure is to combine the urban and rural residents’ medical insurance and let them be managed at the provincial level. In this way, rural residents could reimburse their medical expenditure within the province regardless of the hospital they seek for medical care. Removing obstacles to medical reimbursement would be of great help to the functioning of the New Rural Cooperative Medical Scheme and would improve the health of rural residents to a great extent.

### 4.3. Policy Implementation: Who Will Be Responsible for Managing Medical Reimbursement in China?

Under the context of the current medical reimbursement system, the management center of NCMS in each administrative region is responsible for managing medical reimbursement expenditure and checking rural residents’ reimbursement eligibility. With respect to the implication reimbursement policy based on rural residents’ accessibility, another issue for central or local governments is that they must determine which institution will be responsible for managing medical reimbursement in China. Under the context of the current medical reimbursement system, the reimbursement boundary merely refers to the prefectural boundary. However, when using 2SFCA to delimit the reimbursement region, the reimbursement boundary will be adjusted based on rural residents’ accessibility, as delimiting the reimbursement region and adjusting the reimbursement boundary are not the mandate of the management center of NCMS. More importantly, as it is not a professional institution associated with surveying and mapping, the central or local governments should assign a new institution to do this job and it may be related to the bureau of land and natural resources. In addition, with respect to checking the reimbursement eligibility of rural residents, local governments also need the help and cooperation of other departments, such as the bureau of human resources and social security, bureau of finance, and health commission. As these government sectors have the same administrative level, it is hard to push forward the reform in the specific political context of China. Therefore, the reform should be led by the higher levels of government administration that can organize, manage, and command these departments or be realigned by policy entrepreneurship. A previous study argued that the malleability of rigid institutions can be considerably increased by the active maneuvering of entrepreneurial agents [53]. In any case, if the state wants to address reimbursement inconvenience and grant rural residents the right to freely choose their designated hospitals, the process of reform may be relatively easy and simple.

## 5. Conclusions

By calculating the accessibility ratios of 92 Grade-A hospitals and 313 Grade-B hospitals based on the 2SFCA method, this study investigated the medical accessibility of 1,320,438 residential points based on 30,187 villages in rural Hubei Province. The results indicate that there is significant inequality in access to healthcare among diverse rural residential points in Hubei Province. Furthermore, the current medical reimbursement system, which is divided by prefectural administrative regions, causes reimbursement inconvenience for a substantial proportion of rural residents. Wuhan enjoys the greatest access to healthcare among all prefecture-level administrative areas assessed. Contrastingly, the majority of rural residents from other prefecture-level administrative regions face the difficulties of low medical accessibility and, thus, reimbursement inconvenience caused by the divisions within the medical reimbursement system.

There is obvious spatial disparity between the catchment area of different grades of medical centers and prefecture-level administrative boundaries. For Grade-A hospital services, 28.62% of villages and 13.78% of residential points suffer from reimbursement inconvenience in rural Hubei Province. For Grade-B hospital services, a relatively small number of rural residents cannot reimburse their medical expenses conveniently. Our study shows that the spatial reimbursement boundary should be readjusted. Therefore, we suggest that local governments should delineate the spatial region of medical reimbursement for rural residents according to assessments of their spatial accessibility to different grades of medical centers beyond merely prefecture-level boundaries.

## Figures and Tables

**Table 1:** Descriptive characteristics of hospitals in China.

Row	Hospital Grade	Service Radius	Scale of Hospital	Technical Level
(1)	Grade-A hospital (Sanji hospital)	Mostly within prefecture-level administrative area	More than 501 hospital beds	High
(2)	Grade-B hospital (Erji hospital)	Mostly within county-level administrative area	101–500 hospital beds	Medium
(3)	Grade-C hospital (Yiji hospital)	Mostly within township-level administrative area	Fewer than 100 hospital beds	Low

Data source: Authors’ collection and one land-use/land-cover vector format map (LULC).

**Table 2:** Descriptive statistics of accessibility ratios of rural residents in Hubei Province.

Row	Hospital Grade	Observations (1)	Average (2)	SD (3)	Minimum (4)	Maximum (5)
(1)	Grade-A hospital	30,187	0.22	0.41	0	1.27
(2)	Grade-B hospital	30,187	0.30	0.44	0	2.74
(3)	All	30,187	0.52	0.72	0	3.96

Data source: Authors’ collection and LULC.

**Table 3:** Descriptive statistics of mean accessibility ratios at the prefecture level and hospital level.

Row	Prefecture-Level Administrative Area	Grade-A Hospital (2)	Grade-B Hospital (3)	Both (4)
(1)	Ezhou	0.93	0.76	1.70
(2)	Enshi	0.01	0.02	0.02
(3)	Huanggang	0.15	0.24	0.39
(4)	Huangshi	0.19	0.39	0.58
(5)	Jingmen	0.03	0.10	0.13
(6)	Jingzhou	0.05	0.23	0.28
(7)	Qianjiang	0.06	0.15	0.21
(8)	Shenlongjia	0	0.11	0.11
(9)	Shiyan	0.03	0.15	0.18
(10)	Suizhou	0.02	0.21	0.23
(11)	Tianmen	0.06	0.28	0.34
(12)	Wuhan	1.12	0.78	1.90
(13)	Xiantao	0.11	0.33	0.45
(14)	Xianning	0.07	0.20	0.27
(15)	Xiangyang	0.04	0.50	0.53
(16)	Xiaogan	0.47	0.38	0.86
(17)	Yichang	0.04	0.06	0.10

Data source: Authors’ collection and LULC. Note: Hubei Province has 12 prefecture-level cities and one autonomous prefecture, including three sub-prefecture cities, and one forest region. Since there is a unique administrative division of Hubei Province and incomparability between prefecture-level administrative area and sub-prefecture administrative area, we set the number of prefecture-level administrative areas in Hubei Province as 13 when we computed the service radii of hospitals, but we still made detailed descriptive statistics in Table 3 and Table 4 for other administrative areas.

**Table 4:** Reimbursement inconvenience proportions per prefecture.

Row	Prefecture-Level Administrative Area	Grade-A Hospital		Grade-B Hospital	
		Percentage (Village) (1)	Percentage (Residential Point) (2)	Percentage (Village) (3)	Percentage (Residential Point) (4)
(1)	Ezhou	12.46	12.14	73.94	72.61
(2)	Enshi	0	0	0.45	0.28
(3)	Huanggang	40.81	34.62	12.14	8.01
(4)	Huangshi	72.90	67.47	6.89	7.93
(5)	Jingmen	61.51	53.81	8.73	6.82
(6)	Jingzhou	34.08	31.22	4.87	2.72
(7)	Qianjiang	59.40	65.03	11.35	13.44
(8)	Shenlongjia	0	0	0	0
(9)	Shiyan	0	0	2.71	2.07
(10)	Suizhou	0	0	0	0
(11)	Tianmen	58.41	59.85	21.28	20.69
(12)	Wuhan	4.65	4.55	4.78	5.01
(13)	Xiantao	84.93	88.36	6.66	5.33
(14)	Xianning	22.48	20.37	4.20	1.05
(15)	Xiangyang	1.27	2.05	1.27	2.22
(16)	Xiaogan	65.14	43.03	17.66	22.40
(17)	Yichang	0.72	0.50	2.17	0.44
(18)	Total	28.62	13.78	7.79	3.34

Data source: Authors’ collection and LULC.
